# Diabetic Ketoacidosis With Acute Metabolic Pancreatitis: Two Serious Cases

**DOI:** 10.7759/cureus.20987

**Published:** 2022-01-06

**Authors:** Fatima Zahra Rhmari Tlemçani, Hanane Delsa, Saloua Elamari, Fedoua Rouibaa, Asma Chadli

**Affiliations:** 1 Endocrinology, Diabetes, and Metabolism, Faculty of Medicine, Mohammed VI University of Health Sciences (UM6SS) / Cheikh Khalifa International University Hospital, Casablanca, MAR; 2 Gastroenterology and Hepatology, Faculty of Medicine, Mohammed VI University of Health Sciences (UM6SS), Casablanca, MAR; 3 Endocrinology, Faculty of Medicine, Mohammed VI University of Health Sciences (UM6SS), Casablanca, MAR; 4 Gastroenterology and Proctology, Mohammed VI University of Health Sciences (UM6SS) / Cheikh Khalifa International University Hospital, Casablanca, MAR; 5 Endocrinology, Diabetology, Metabolic Disease, and Nutrition, Mohammed VI University of Health Sciences (UM6SS), Casablanca, MAR

**Keywords:** hypertriglyceridemia, metabolic pancreatitis, severe acute pancreatitis, ketoacidosis, diabetes

## Abstract

Hypertriglyceridemia is a rare cause of acute pancreatitis (AP), occupying approximately 7% of cases. The triad of acute pancreatitis, hypertriglyceridemia, and diabetes is a rare event, with a higher death rate.

We describe two cases of severe acute metabolic pancreatitis discovered in diabetic ketoacidosis. For both patients, all other causes of AP were excluded (including gallstones, hypercalcemia, drugs, and alcohol). A laboratory workup revealed elevated lipasemia (more than three times the normal) and hypertriglyceridemia. Abdominal computed tomography confirmed the diagnosis of severe acute pancreatitis. Fasting, fluid resuscitation, and insulin therapy were initiated in the intensive care unit with good clinical results and progressive improvement in metabolic disorders.

## Introduction

Acute pancreatitis (AP) is most often caused by gallstones or alcohol in 60%-75% of cases. This medical emergency continues to increase in prevalence due to the emergence of other etiologies. Only 5%-10% of AP is caused by metabolic conditions like hypertriglyceridemia, diabetes, hypercalcemia, Wilson's disease, and porphyria [1]. Episodes of metabolic pancreatitis tend to be more severe and require careful management of the underlying metabolic abnormalities.

We describe two cases of severe acute metabolic pancreatitis discovered in the context of diabetic ketoacidosis. For both patients, all other causes of AP were excluded (including biliary gall stones, hypercalcemia, drugs, and alcohol).

## Case presentation

Observation 1

A 44-year-old woman was admitted to the emergency room for acute epigastric pain radiating to the periumbilical region, accompanied by vomiting. Her past medical history included hypertriglyceridemia treated by fenofibrate, and type 2 diabetes, managed for seven years with oral antidiabetics (glimepiride 3 mg per day and metformin 2 g per day). There was no personal history of alcohol use or tobacco. The patient was stable; her clinical examination revealed dyspnea with abdominal tenderness. A decompensation of her diabetes was suspected and confirmed: capillary blood glucose level of 4 g/L (normal reference: 0.6 to 1 g/L) and ketonuria of three crosses.

The results of blood sampling showed a high lipasemia (11 times the upper limit) and biological inflammatory syndrome: high serum level of C-reactive protein (CRP) = 82.18 mg/L and hyperleukocytosis of 12390 cells/mm^3^ with a predominance of neutrophils. The rest of the biological results showed hypertriglyceridemia at 28.14 g/L (normal reference: less than 1.50), low alkaline reserves at 14.1 mmol/L (normal reference: 22-29), and hyponatremia at 124 mmol/L (normal reference: 136-145).

**Figure 1:**
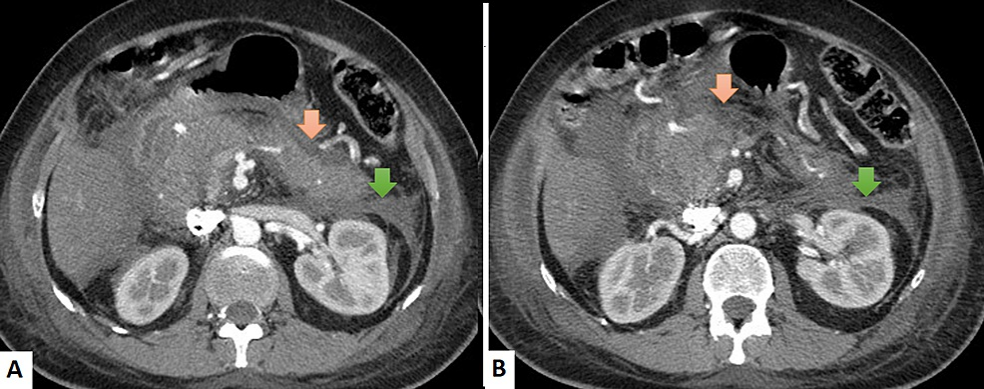
Contrast-enhanced CT images in axial plans showing acute pancreatitis peripancreatic fat standing (orange arrow), fluid collection associated with perinephric fascial thickening (green arrow) evocative of severe pancreatitis (A and B)

An abdominal computed tomography (CT) scan revealed signs of acute pancreatitis (grade E) according to the Balthazar score: peripancreatic fat infiltration with fluid collection (Figure 1). Magnetic resonance cholangiopancreatography (MRCP) confirmed the absence of occult common bile duct stones.

The patient was quickly admitted to the intensive care unit (ICU) where her treatment involved fasting, fluid resuscitation, insulin therapy, and administration of analgesic treatment (morphine and nefopam hydrochloride). The patient’s condition improved to a complete recovery and was transferred to our department after one week. History revealed that two months before admission, her hemoglobin A1c level was 11.5% (normal reference: 4.5-6), confirming the previous glycemic imbalance, the patient was also overweight with a body mass index (BMI) of 27.6 kg/m^2^ (height of 1.65 cm with a weight of 75 kg). The therapeutic protocol allowed the correction of the glycemic imbalance, especially diabetic ketosis. Unsaturated oils (omega 3.6.9) were also administered.

The clinical and laboratory outcomes were favorable: the disappearance of the pain with an improvement in the laboratory results (Table 1). The triglyceride level gradually decreased until normalization (the last level is 1.56 g/L), with radiological improvement on follow-up imaging at one month.

**Table 1:** Evolution of the biochemical characteristics of the cases CRP: C-reactive protein

BIOCHEMICAL ASSESSMENT	TG (g/L)		CRP (mg/L)		White Blood Cells (/mm^3^)	
	Case 1	Case 2	Case 1	Case 2	Case 1	Case 2
1st day	28,14	12,92	82,18	557,97	12390	17900
7th day	4,17	1,90	104,98	162,12	11240	13020
10th day	1.56	2,05	76,76	123,87	8400	11580
Reference values	< 1.50		< 5		4000 - 11000	

Observation 2

**Figure 2:**
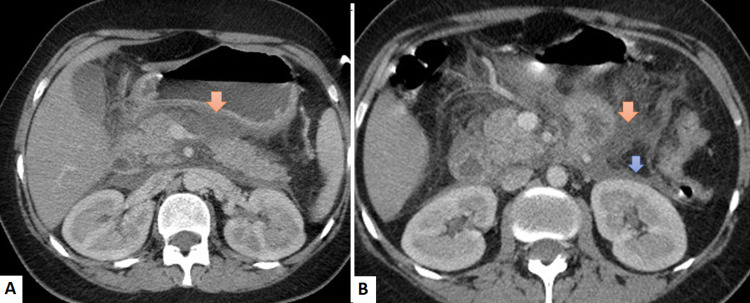
Contrast-enhanced computed tomography images revealing acute pancreatitis A: Diffusely edematous pancreas with peripancreatic fluid collection (orange arrow) B: Perinephric fascia thickening (blue arrow)

A 19-year-old girl presented with a history of type 1 diabetes for seven years on basal-bolus insulin and without a personal or family history of hypertriglyceridemia. The patient was admitted to the emergency room for acute epigastric abdominal pain and a confusional state in the context of vomiting. On clinical examination, we found the patient was hemodynamically stable with dyspnea, fever, and vulvovaginal mycosis associated with decompensation of her diabetes (capillary blood glucose level of 5.9 g/L and ketonuria of three crosses). Her anthropometric measurements confirmed the overweight with a BMI of 28 kg/m^2^ (height of 1.56 cm and weight of 68 kg). Full laboratory assessment revealed high lipasemia (10 times the upper limit), hypertriglyceridemia at 12,92 g/L, acidosis at 10.5 mmol/L (normal reference: 22-29), hyponatremia at 121 mmol/L, and a biological inflammatory syndrome with a CRP of 557.97 mg/L, and hyperleukocytosis of 17900 cells/mm^3^, the infectious assessment showed leukocyturia without bacteriuria. CT scan revealed peripancreatic fluid collections suggestive of severe acute pancreatitis (Figure 2), confirmed by MRCP without others causes of pancreatitis.

Fasting, fluid resuscitation, and insulin therapy were initiated in the intensive care unit, with good clinical outcomes. She was treated with continuous insulin infusion and intravenous antibiotics (2 g of third-generation cephalosporins associated with 1.5 g of metronidazole per day with fluconazole as an antifungal medication).

At admission, the biochemical assessment revealed a glycemic imbalance with a hemoglobin A1c level of 12.7%. One week later, the neurological and digestive signs resolved completely, and the patient was entirely independent without sequelae. The treatment protocol allowed the management of diabetic ketosis. In our patient, unsaturated oils (omega 3.6.9) were administered. The level of triglycerides gradually decreases until normalization (Table 1), without any complications. At the pancreatic level, control imaging showed a clear improvement of the lesions.

## Discussion

Metabolic pancreatitis is an uncommon cause of acute pancreatitis. Occasionally, diabetic ketoacidosis can cause it, complicated by hypertriglyceridemia (HTG), which requires intensive care management. Nevertheless, the connection between diabetic ketoacidosis, hypertriglyceridemia, and acute pancreatitis has rarely been reported. The triad of diabetic ketoacidosis (DKA), HTG, and AP is rarely seen in patients [2].

Concerning the mechanisms of the pathophysiology, there is a bidirectional relationship between dysglycemia and pancreatitis [3]. Acute pancreatitis induces a systemic inflammation that could be responsible for a dysfunction of beta cells and, consequently, an insulin deficiency associated with insulin resistance [4]. The prospective study of Nair et al. showed that diabetic ketoacidosis is a risk factor for hypertriglyceridemia-induced acute pancreatitis (HTGP); 11% of their patients diagnosed with diabetic ketoacidosis had acute pancreatitis [5].

On the other hand, with regard to the pathophysiology of HTGP in acute pancreatitis, the very high concentration of free fatty acids (FFA) exceeds the binding capacity of plasma albumin. The FFA will self-aggregate and then form micellar elements with detergent properties that destroy platelets, acinar, and vascular endothelial cells, inducing a cascade of pancreatic injury.

A theory suggested that increased levels of chylomicrons may lead to plasmatic hyperviscosity as the primary mechanism of HTG-induced PA by provoking ischemia and acidosis in pancreatic capillaries [6-7]. The causes of HTG can be classified into two categories of disorders: primary (genetic) and secondary. Recently, Zixi Huang et al. described three new heterozygous missense mutations: c.160G > C (p.Glu54Gln) in CILP2, c.12614C > T (p.Pro4205-Leu) in APOB, and c.1199C > A (p.Ala400Glu) in peptidase D in patients with the triad of AP + HTG + DKA [8].

The diagnosis of acute pancreatitis was suggested in our patients suffering from acute epigastric pain despite the confirmation of ketoacidosis and confirmed by the lipasemia associated with the CT scan. The clinical presentation of patients with the triad is underdiagnosed because it is similar to those with DKA only.

The large cohort of Simons-Linares CR et al. demonstrated that mortality in patients with the HTG, DKA, and AP triad was significantly higher compared to AP-only patients with a higher risk of complications (acute respiratory distress syndrome shock, sepsis, and so on) [9].

Consequently, we suggest that health professionals should consider acute pancreatitis at the outset when any patient is admitted for AP associated with HTG to improve the prognosis of the triad. In patients presenting with AP, without a significant history of alcohol consumption or gallstones, serum triglyceride level should be measured on admission. When it is greater than 11.3 mmol/l (1000 mg/dl), it is probably the cause of AP [10].

In patients without a significant history of alcohol use or gallstones, serum triglyceride levels should be measured. When it is over 11.3 mmol/l (1000 mg/dl), it is probably the cause of AP.

The management of HTGE patients includes several approaches: intravenous hydration, bowel rest, pain management, reducing TG levels, therapy, and symptomatic care.

Nevertheless, this treatment may not be sufficient in some cases. Indeed, the main objective of treatment, in this case, is to reduce the TG level quickly. It has been suggested that therapeutic plasma exchange (TPE) treatment is an interesting way to lower very high TG levels [11]. It has also been suggested that TPE is the most interesting option for HTG >2000 mg/dl and has also shown excellent results in severe forms of HTGP, according to the Revised Atlanta Classification [12].

Both our patients were admitted to the ICU quickly in order to receive emergency assistance with good outcomes and complete resolution of symptoms. Patient management requires the cooperation of several specialists (gastroenterologists, endocrinologists, radiologists, and emergency doctors); therefore, the creation of standardized protocols to decrease the mortality of this group of patients is essential.

## Conclusions

Diabetic ketoacidosis associated with hypertriglyceridemia-induced acute pancreatitis is an uncommon and serious situation. However, it must be evoked at the outset of diabetic patients presenting with acute epigastric pain. This severe complication was suspected in our patients and confirmed based on clinical presentation, abdominal CT, blood test, and ketonuria. Multidisciplinary care with early treatment improved the prognosis of our patients by avoiding irreversible complications.
